# Relationship between small intestinal bacterial overgrowth and irritable bowel syndrome and the efficacy of rifaximin intervention: a systematic review and meta-analysis

**DOI:** 10.3389/fmicb.2026.1780567

**Published:** 2026-03-10

**Authors:** Huigang Lu

**Affiliations:** Department of Gastroenterology, Children’s Hospital of Soochow University, Suzhou, China

**Keywords:** irritable bowel syndrome, meta-analysis, relationship, rifaximin, small intestinal bacterial overgrowth

## Abstract

**Objective:**

To systematically investigate the pooled prevalence of Small Intestinal Bacterial Overgrowth (SIBO) in patients with Irritable Bowel Syndrome (IBS) compared to healthy individuals and to evaluate the therapeutic efficacy of rifaximin in IBS patients with concomitant SIBO.

**Methods:**

A comprehensive computer-based search was conducted across databases including PubMed and Embase from their inception until December 2025. Relevant cohort studies, case-control studies, and cross-sectional studies were included. Study quality was assessed using the Newcastle-Ottawa Scale (NOS). Meta-analysis was performed using a random-effects model.

**Results:**

A total of 25 studies were included. The pooled risk of SIBO was significantly higher in IBS patients compared to healthy controls (OR = 5.71, 95% CI: 3.45–9.45). The Glucose Hydrogen Breath Test (GBT) subgroup showed a higher odds ratio and lower heterogeneity. Rifaximin treatment achieved a pooled SIBO eradication rate of 59% (95% CI: 0.48–0.68), with the medium-to-high dose group (≥ 1,200 mg/day) showing a slightly superior efficacy compared to the lower-dose group.

**Conclusion:**

A significant association exists between SIBO and IBS. Rifaximin is an effective treatment for IBS patients with concomitant SIBO. The substrate choice for SIBO diagnosis involves a trade-off: while GBT offers greater diagnostic stability and specificity, LBT provides broader sensitivity for distal overgrowth. This evidence-based nuance should guide clinical substrate selection based on the diagnostic priority.

**Systematic review registration:**

INPLASY.COM, identifier NPLASY202630002.

## 1 Introduction

Irritable Bowel Syndrome (IBS) is a highly prevalent disorder of gut-brain interaction with a global footprint. Its clinical presentation is defined by a triad of chronic, recurrent symptoms: Abdominal discomfort or pain, sensations of bloating or distension, and a change in the frequency or form of stools, manifesting as diarrhea (IBS-D), constipation (IBS-C), or a mixed pattern (IBS-M) (Thompson, 2016). A key diagnostic hallmark is the absence of identifiable structural or biochemical abnormalities through routine clinical evaluation. Current epidemiological estimates suggest that IBS impacts approximately one in ten individuals worldwide, with prevalence rates consistently reported within the 10–15% range across diverse populations (Black and Ford, 2020). This high prevalence translates into significant personal suffering, marked reductions in work productivity and daily functioning, and considerable direct and indirect economic costs to healthcare systems, positioning IBS as a major public health concern. The pathogenesis of IBS is multifactorial, with dysregulation of the gut-brain axis, visceral hypersensitivity, and altered gastrointestinal motility being established contributors. In recent years, the role of gut microbial dysbiosis has emerged as a critical area of investigation (Ghoshal et al., 2017; Abraham and Pratap, 2023).

Small Intestinal Bacterial Overgrowth (SIBO) is defined as a pathological increase in the bacterial load (typically > 10^3^ colony-forming units per milliliter) or an alteration in the microbial composition within the small intestine (Silva et al., 2025). Under physiological conditions, the proximal small bowel maintains a relatively low bacterial count. Factors such as impaired intestinal motility, reduced gastric acid secretion, and compromised mucosal integrity can predispose to the colonization and proliferation of bacteria in this region. A growing body of evidence suggests a significant overlap between SIBO and IBS (Ghosh and Jesudian, 2019). The reported prevalence of SIBO in IBS patients varies widely (4–78%), yet consistently exceeds that observed in healthy controls (1–40%), with a notably higher frequency in the diarrhea-predominant (IBS-D) subtype (Ghoshal et al., 2017; Ghoshal et al., 2020). Proposed mechanistic links include excessive gas production (hydrogen, methane) from bacterial fermentation of carbohydrates, leading to bloating and pain, and the potential for bacterial metabolites to disrupt intestinal barrier function and incite low-grade inflammation (Takakura and Pimentel, 2020; Wilk et al., 2025; Calanni et al., 2014). However, considerable heterogeneity exists across studies, partly attributable to the lack of standardized diagnostic criteria for SIBO, with methods like lactulose or glucose breath testing and jejunal aspiration culture yielding variable results.

The management of IBS patients with concomitant SIBO often involves antibiotic therapy. Among the available options, rifaximin has garnered particular attention due to its pharmacological profile. Rifaximin is a non-systemic, broad-spectrum antibiotic derived from rifamycin (Calanni et al., 2014; Pimentel, 2009; Zhuang et al., 2020). It acts locally within the gastrointestinal tract with minimal systemic absorption, resulting in a favorable safety and tolerability profile. Clinical trials have demonstrated that rifaximin can improve global symptoms in 33–92% of IBS patients, achieve SIBO eradication rates as high as 84%, and provide sustained relief for up to 10 weeks post-treatment (Pimentel, 2009; Zhuang et al., 2020; Lauritano et al., 2009; Pimentel et al., 2004). Furthermore, compared to systemic antibiotics like metronidazole or levofloxacin, rifaximin is associated with a lower incidence of adverse effects and a negligible risk of inducing significant bacterial resistance (Lauritano et al., 2009).

Given the ongoing debate regarding the SIBO-IBS association and to consolidate evidence on a targeted therapeutic strategy, this study aims to conduct a systematic review and meta-analysis. Our objectives are twofold: (1) to quantitatively synthesize data on the pooled prevalence of SIBO in IBS patients compared to healthy individuals, and (2) to evaluate the efficacy of rifaximin in terms of symptom response and SIBO eradication rates in IBS patients diagnosed with SIBO. The findings are intended to inform clinical decision-making and optimize therapeutic approaches for this challenging patient population.

## 2 Methods

### 2.1 Study design

This is a systematic review and meta-analysis conducted to evaluate the association between SIBO and IBS and to assess the therapeutic efficacy of rifaximin in IBS patients with confirmed SIBO.

### 2.2 Search strategy

To ensure a thorough and systematic retrieval of pertinent evidence, an extensive search was conducted across multiple major electronic databases. The search encompassed PubMed, Embase, the Cochrane Central Register of Controlled Trials (CENTRAL), and Web of Science Core Collection, covering the period from each database’s inception through December 2025. A meticulously developed search strategy was implemented, utilizing both controlled vocabulary (e.g., MeSH in PubMed, Emtree in Embase) and relevant free-text keywords to maximize sensitivity and specificity. Core search concepts included terms such as “Irritable Bowel Syndrome” (and its abbreviation “IBS”), “Small Intestinal Bacterial Overgrowth” (“SIBO”), the antibiotic “rifaximin,” and diagnostic methodologies like “breath tests” (including “hydrogen breath test”). To further minimize the risk of omitting relevant studies, a manual examination of the bibliographies of all articles selected for full-text review, as well as those of key systematic reviews and meta-analyses identified during the search, was performed to locate any additional publications meeting the eligibility criteria.

### 2.3 Eligibility criteria

#### 2.3.1 Study design

Observational studies (cohort, case-control, or cross-sectional) investigating the SIBO-IBS association, and intervention studies (including randomized controlled trials and open-label studies) reporting on rifaximin treatment for IBS/SIBO.

#### 2.3.2 Participants

Adult patients (typically ≥ 18 years) diagnosed with IBS according to established clinical criteria. While the majority of included studies strictly enrolled adults and utilized Rome III or IV criteria, certain pediatric or adolescent cohorts (e.g., Scarpellini et al., 2009) and studies employing Rome I, II, Manning, or symptom-based clinical diagnoses were also included to ensure a comprehensive synthesis of landmark data, acknowledging these variations as potential sources of methodological heterogeneity.

For the control arm, “healthy individuals” were defined as asymptomatic volunteers with no history of chronic gastrointestinal disorders. Preference was given to studies that screened controls to exclude active GI symptoms, though unscreened asymptomatic individuals were accepted where explicitly described by primary authors.

#### 2.3.3 Exposure/diagnosis

For association studies, SIBO diagnosis was required to be based on hydrogen/methane breath testing (lactulose or glucose) or jejunal aspirate culture.

#### 2.3.4 Intervention/outcome

For intervention studies, treatment with rifaximin (any dose/duration) was required. Primary outcomes of interest were: (1) Prevalence/odds of SIBO in IBS vs. controls; (2) SIBO eradication rate post-rifaximin treatment (based on negative follow-up breath test); (3) Global IBS symptom improvement rate.

#### 2.3.5 Exclusion criteria

Reviews, case reports, conference abstracts, editorials, and animal studies; studies with overlapping patient populations or duplicate publications; studies reporting incomplete data or from which effect estimates could not be extracted.

### 2.4 Data extraction

To ensure objectivity and minimize errors in data collection, the extraction process was independently conducted by two researchers. Each reviewer utilized a pre-designed, piloted data extraction sheet to systematically record information from the included studies. Any disagreements or inconsistencies identified between the two reviewers’ extracted data were addressed through a consensus-building discussion. If consensus could not be reached, a third senior researcher was consulted for arbitration and final adjudication. The specific data points collected for analysis encompassed: study identification details (first author, publication year, country of origin), methodological characteristics (study design, total sample size), participant specifics (IBS diagnostic criteria and subtype classification), SIBO diagnostic parameters (methodology and positivity thresholds), intervention details for treatment studies (rifaximin dosage and treatment duration), and all pertinent outcome data (e.g., counts of SIBO-positive individuals in case and control groups, post-treatment eradication rates, and clinical symptom response rates).

To detect and manage potential overlapping populations or duplicate cohorts—particularly those originating from the same research centers and overlapping time periods—study metadata including author names, institutional affiliations, recruitment dates, and sample sizes were cross-referenced. In instances of confirmed or suspected redundancy, only the publication providing the largest sample size or the most comprehensive outcome reporting was retained for analysis to prevent the inflation of effect estimates.

### 2.5 Evaluation of study quality

A rigorous assessment of the methodological rigor and potential for bias in the included studies was performed. For observational studies (e.g., case-control, cross-sectional), quality was appraised using the Newcastle-Ottawa Scale (NOS). This tool provides a structured evaluation across three critical domains: the selection of study groups, the comparability of groups, and the ascertainment of either the exposure (for case-control studies) or outcome (for cohort studies). Studies achieving a total score of 7 or higher out of a possible 9 points were deemed to be of high methodological quality. For interventional studies, particularly randomized controlled trials (RCTs), the planned instrument for bias assessment was the Cochrane revised Risk of Bias tool (RoB 2), which evaluates bias across five specific domains pertaining to the randomization process, deviations from intended interventions, missing outcome data, outcome measurement, and selection of the reported result.

### 2.6 Statistical analysis

For the association between IBS and SIBO, the pooled odds ratio (OR) with a 95% confidence interval (CI) was calculated, comparing the odds of SIBO in IBS patients versus healthy controls. For treatment efficacy, the pooled event rate (ER) for SIBO eradication was computed. Due to the heterogeneous metrics used to report symptoms (e.g., IBS-SSS, VAS scores, or Likert scales), a structured narrative synthesis was performed for patient-centered outcomes—including abdominal pain, bloating, stool patterns, and quality of life (IBS-QOL)—rather than a formal meta-analysis. In recognition of the expected variability in patient populations, study designs, and measurement approaches among the included investigations, a DerSimonian and Laird random-effects model was selected *a priori* as the primary analytical framework for all meta-analytic calculations. This model incorporates an estimate of between-study variance (τ^2^), providing a more conservative and generalized summary effect estimate suited for heterogeneous data. To quantify the degree of inconsistency across study results, the I^2^ statistic was employed. An *I*^2^ value exceeding 50% was interpreted as representing substantial statistical heterogeneity, warranting further exploration of its potential sources.

To investigate the origins of heterogeneity and to address specific clinical questions, pre-specified subgroup analyses were planned. These analyses aimed to examine potential effect moderators, including clinical presentation (IBS-D, IBS-C, IBS-M), diagnostic methodology for SIBO (glucose versus lactulose substrate breath tests), geographical region of study origin, and treatment duration (e.g., 10 days vs. 14 days). The stability and reliability of the primary pooled effect estimates were interrogated through sensitivity analyses. This was performed by iteratively removing individual studies from the analysis and recalculating the summary statistic to determine if any single study exerted a disproportionate influence on the overall result. The potential for publication bias was evaluated both graphically and statistically. Funnel plots were visually inspected for asymmetry. For meta-analyses comprising ten or more studies, Egger’s linear regression test was applied to provide a formal quantitative assessment of funnel plot asymmetry. All data synthesis and statistical computations were executed utilizing R statistical software (Version 4.3.0), with specific analyses performed using the “meta” and “metaphor” packages.

## 3 Results

### 3.1 Study selection

**FIGURE 1:**
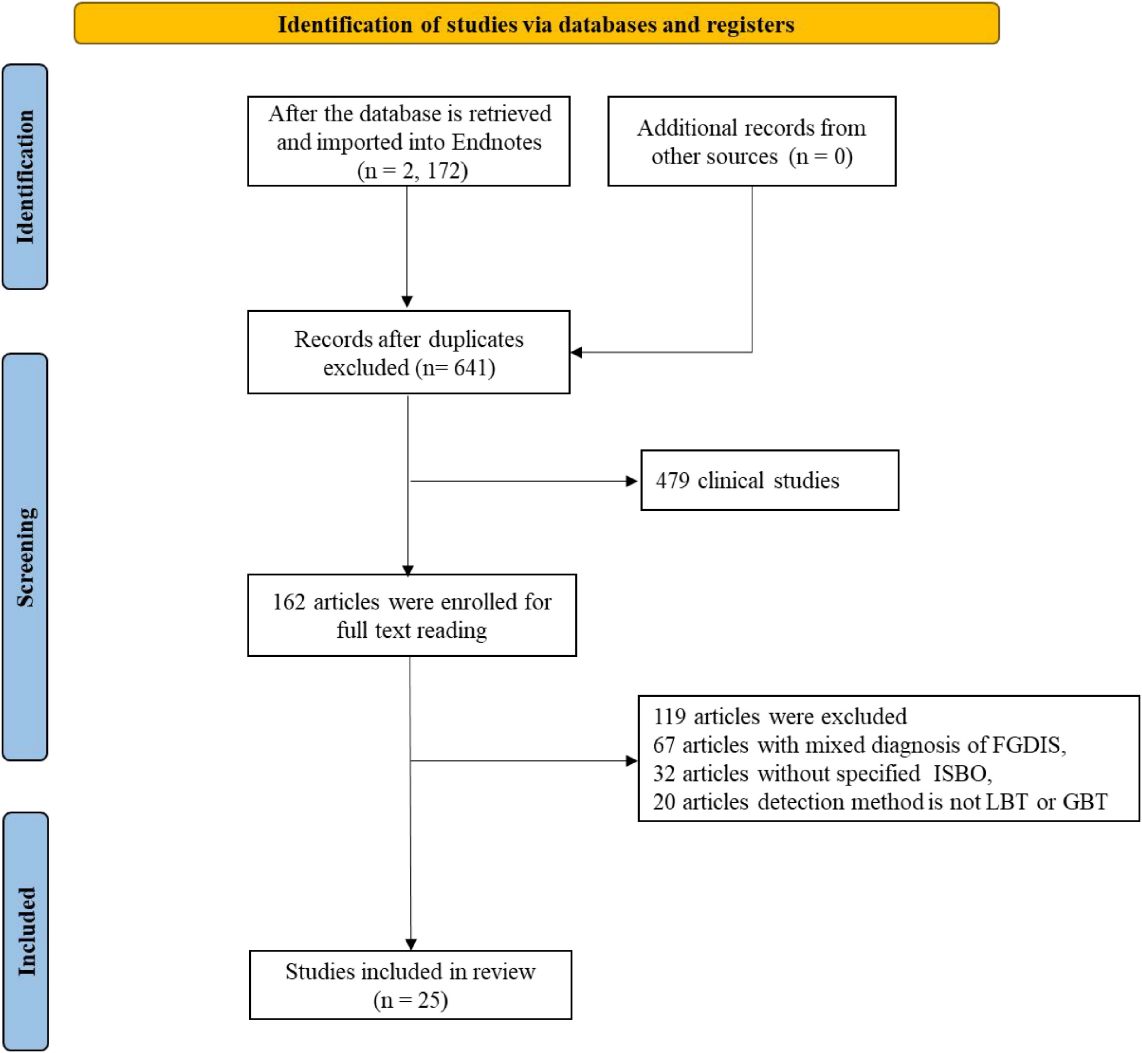
Flow diagram of literature screening and selection.

The systematic search across the specified databases initially identified a total of 2,172 potentially relevant citations. Following the removal of 1,531 duplicate entries, a pool of 641 unique records proceeded to the first phase of screening. These records were independently evaluated based on their titles and abstracts against the predefined eligibility criteria, leading to the exclusion of 479 publications. Common reasons for exclusion at this stage included non-relevant study designs (e.g., reviews, commentaries, animal or *in vitro* studies) and studies clearly focused on populations or conditions outside the scope of this review. Subsequently, the full-text versions of the remaining 162 articles were retrieved and subjected to a detailed eligibility assessment. Among these, 137 articles were excluded, primarily due to: incomplete or irreconcilable outcome data (*n* = 32), inclusion of patients with comorbid organic gastrointestinal diseases (*n* = 61), inappropriate study design (*n* = 29), or use of non-standard SIBO diagnostic criteria (*n* = 15). Consequently, 25 studies met the inclusion criteria and were incorporated into the qualitative and quantitative synthesis. Among these, 1 study provided data for both the SIBO-IBS association and rifaximin treatment outcomes, 16 studies contributed solely to the association analysis, and 8 studies contributed solely to the rifaximin efficacy analysis (Figure 1).

### 3.2 Study characteristics

A total of 15 case-control studies evaluating the association between IBS and SIBO were included in the meta-analysis. As the study by Rana et al. (2012) utilized two different diagnostic methods (LBT and GBT), it was analyzed as separate subgroups. These studies were conducted across Asia, Europe, and the Americas, encompassing 1,765 IBS patients and 1,008 healthy controls. The diagnostic criteria for IBS varied among the studies, including Rome I, II, III, IV, and the Manning criteria. The inclusion of these diverse criteria was necessary to capture the full chronological breadth of research on the IBS-SIBO association, as many high-quality early studies predate the current Rome IV standards. SIBO diagnosis was established using either lactulose hydrogen breath tests (LBT) or glucose hydrogen breath tests (GBT). The quality of all included observational studies was moderate to high (Table 1).

**TABLE 1:** Characteristics of studies investigating the association between IBS and SIBO.

Author	Year	Region	IBS, N	Criteria for IBS	Controls, N	Diagnosis of SIBO	SIBO in IBS	SIBO in controls, n	Nos
Pimentel et al. (2004)	2004	United States	111	Rome I	15	LBT	93	3	7
Scarpellini et al. (2009)	2009	Italy	43	Rome II	56	LBT	28	4	8
Park et al. (2009)	2009	Korea	38	Rome II	12	LBT	7	1	7
Park et al. (2010)	2010	Korea	76	NA	40	LBT	34	16	7
Zhao et al. (2014)	2014	China	89	Rome III	13	LBT	35	1	7
Lupascu et al. (2005)	2005	Italy	65	Rome II	102	GBT	20	4	7
Rana et al. (2008)	2008	India	225	Rome II	100	GBT	25	1	6
Parodi et al. (2009)	2009	Italy	130	Rome III	70	GBT	21	3	7
Ghoshal et al. (2010)	2010	India	129	Manning	51	GBT	11	1	7
Rana et al. (2012)	2011	India	175	Rome II	150	LBT	60	45	8
						GBT	11	1	
Sachdeva et al. (2011)	2011	India	59	Rome III	37	GBT	14	1	6
Abbasi et al. (2015)	2015	Iran	107	Rome III	107	GBT	40	13	7
Moraru et al. (2014)	2014	Romania	331	Rome III	105	GBT	105	7	6
Yu et al. (2021)	2021	China	127	Rome III	90	LBT	101	26	7
Wu et al. (2019)	2019	China	60	Rome IV	60	LBT	31	10	7

For the evaluation of rifaximin’s efficacy, 9 clinical studies were included, conducted in regions including Asia, Europe, North and South America, and Africa. These studies comprised a total of 292 IBS patients diagnosed with concomitant SIBO. The IBS diagnostic criteria used in these studies were Rome II, III, IV, or symptom-based clinical diagnosis. SIBO was confirmed via LBT or GBT. The rifaximin dosage ranged from 600 to 1,650 mg per day, administered over a treatment duration of 10 days to 2 weeks. All studies reported the number of SIBO-positive cases before and after treatment. Detailed baseline characteristics and intervention protocols for each study are summarized in Table 2.

**TABLE 2:** Characteristics of studies evaluating rifaximin for the treatment of IBS with concomitant SIBO.

Author	Year	Region	Criteria for IBS	Diagnosis of SIBO	Rifaximin	Duration	SIBO pre-treatment	SIBO post-treatment
Zhuang et al. (2020)	2020	China	Rome IV	LBT	800 mg/day	2 Weeks	45	25
Moraru et al. (2013)	2013	Romania	Rome III	GBT	1,200 mg/day	2 Weeks	8	1
Cuoco and Salvagnini (2006)	2006	Italy	Symptoms-based	GBT	1,200 mg/day	2 Weeks	23	4
Scarpellini et al. (2013)	2013	Rome	Rome II	LBT	600 mg/day	1 Week	33	12
Peralta et al. (2009)	2009	Italy	Rome II	LBT	1,200 mg/day	1 Week	54	26
Parodi et al. (2009)	2009	Italy	Rome III	GBT	1,200 mg/day	10 Days	24	7
von Muhlenbrock et al. (2025)	2025	Chile	Rome IV	LBT	800 mg/day	10 Days	27	11
García-Cedillo et al. (2025)	2025	Mexico	Rome IV	LBT	1,200 mg/day	2 Weeks	25	17
Barkin et al. (2019)	2019	US	Rome IV	GBT	1,650 mg/day	2 Weeks	53	25

### 3.3 Association between SIBO and IBS

**FIGURE 2:**
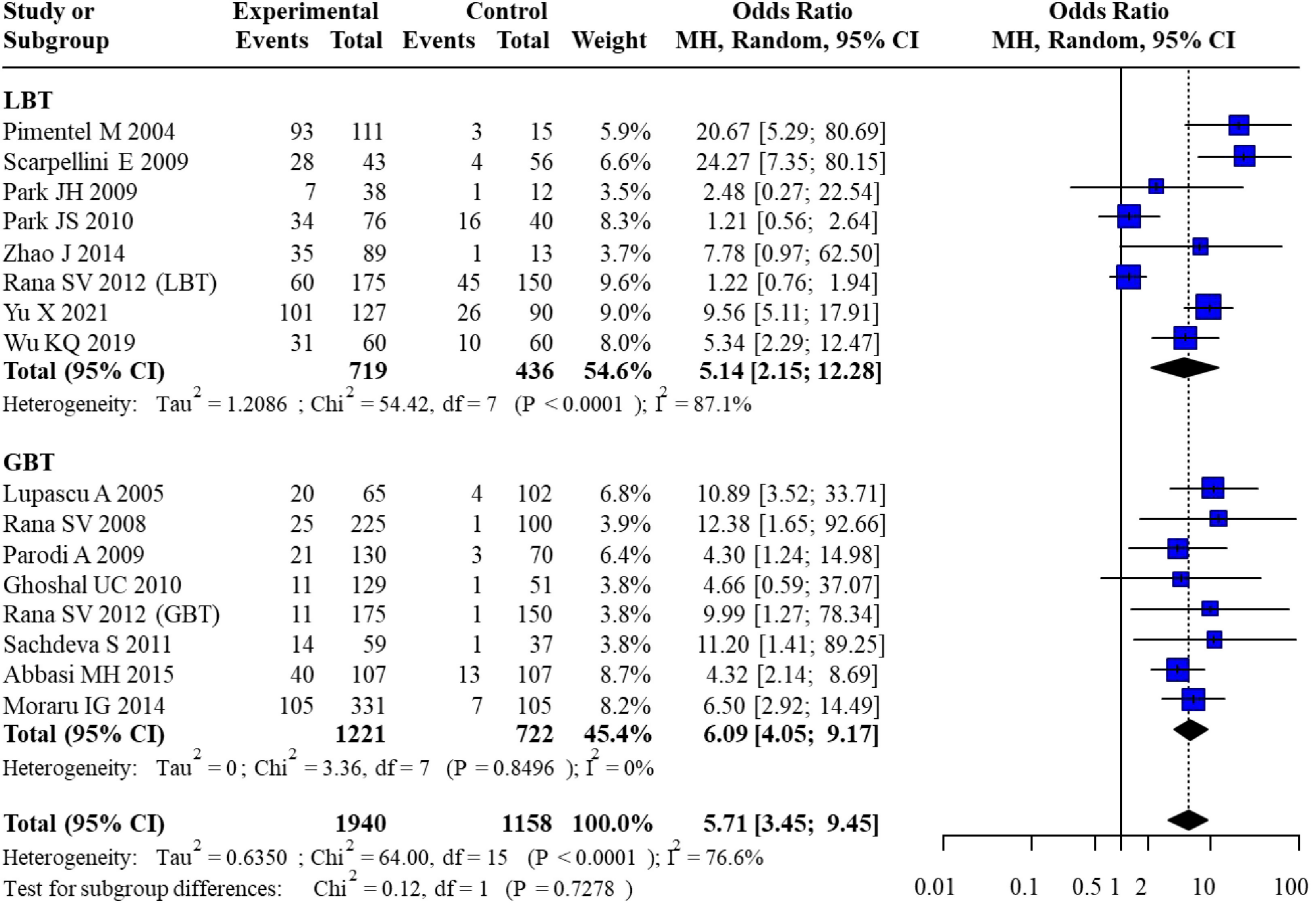
Forest plot for the association between SIBO and IBS.

The pooled analysis revealed a significant association between IBS and SIBO across all studies. In the subgroup of studies using LBT for SIBO diagnosis, the odds ratios (ORs) for SIBO risk in IBS patients compared to healthy controls ranged from 1.22 to 24.27. The pooled OR for this subgroup was 5.14 (95% CI: 2.15–12.28). In the GBT subgroup, individual study ORs ranged from 4.32 to 12.38, yielding a pooled OR of 6.09 (95% CI: 4.05–9.17). The overall pooled OR across all diagnostic methods was 5.71 (95% CI: 4.05–9.45), indicating that IBS patients had nearly six times higher odds of having SIBO compared to healthy individuals in Figure 2.

**FIGURE 3:**
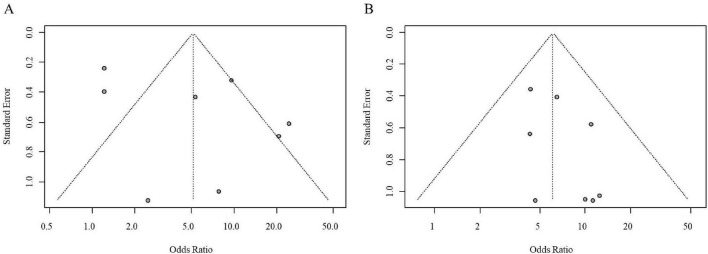
Funnel plots for publication bias assessment in the association analysis between SIBO and IBS (**A**, LBT subgroup; **B,** GBT subgroup).

Assessment of publication bias using funnel plots suggested a relatively symmetrical distribution for studies in the LBT subgroup in Figure 3A. However, in the GBT subgroup in Figure 3B, the funnel plot indicated a potential asymmetry with a greater number of studies plotted on the right side and a higher density of studies near the bottom, hinting at possible publication bias.

**FIGURE 4:**
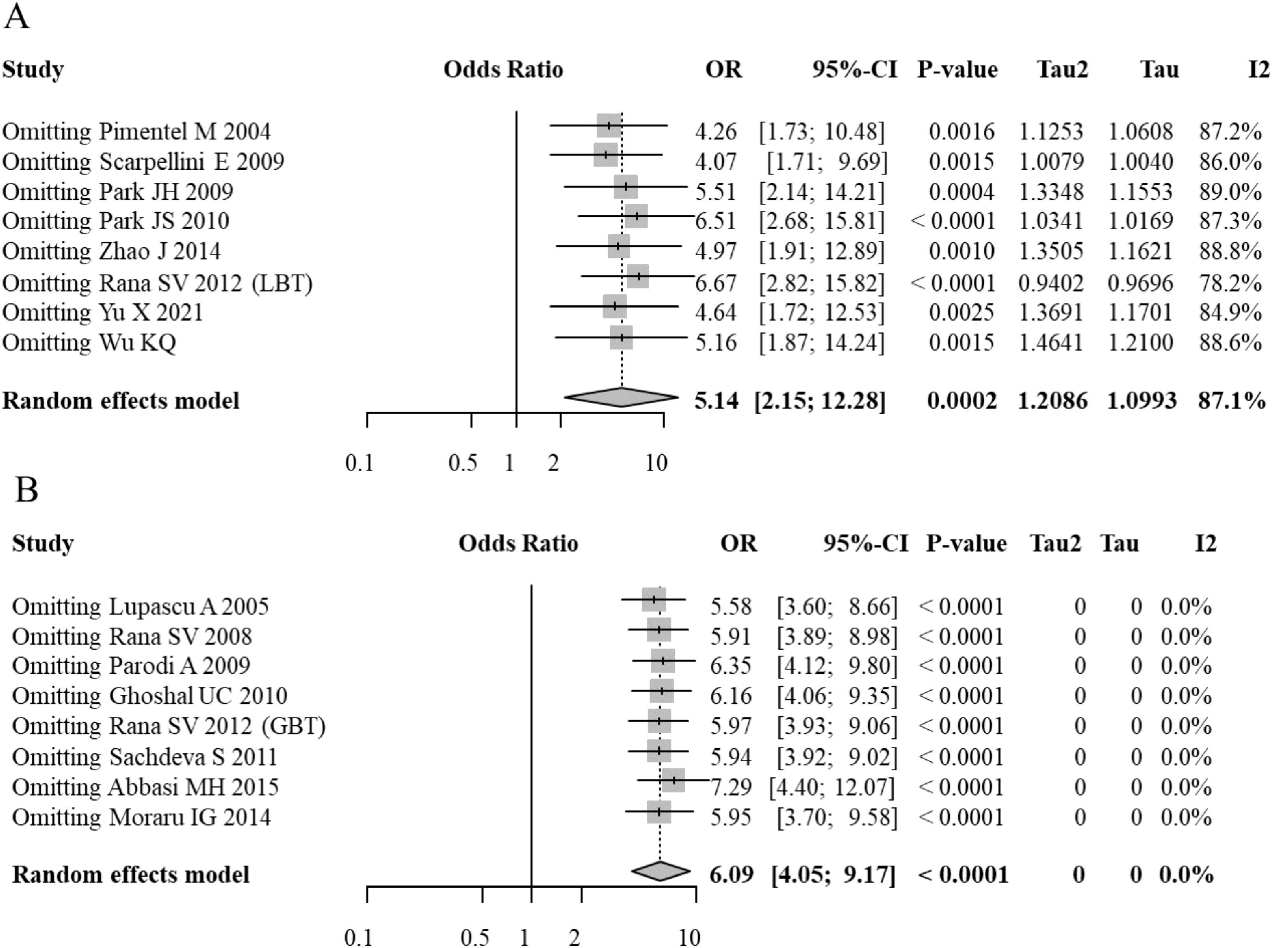
Sensitivity analysis (leave-one-out) forest plots for the association between SIBO and IBS (**A**, LBT subgroup, **B**, GBT subgroup).

In the LBT subgroup, the pooled OR ranged from 4.07 to 6.67 upon exclusion of individual studies, with all 95% CIs remaining above the null value (OR = 1). In the GBT subgroup, the pooled OR varied between 5.58 and 7.29, with confidence intervals consistently excluding unity. While the overall significance of the association was stable in both subgroups, the LBT subgroup exhibited higher heterogeneity across multiple studies, potentially reflecting its variable specificity. Conversely, the GBT subgroup results showed minimal heterogeneity, suggesting higher operational consistency, though this may come at the expense of lower sensitivity for distal SIBO in Figure 4.

### 3.4 Therapeutic efficacy of rifaximin

**FIGURE 5:**
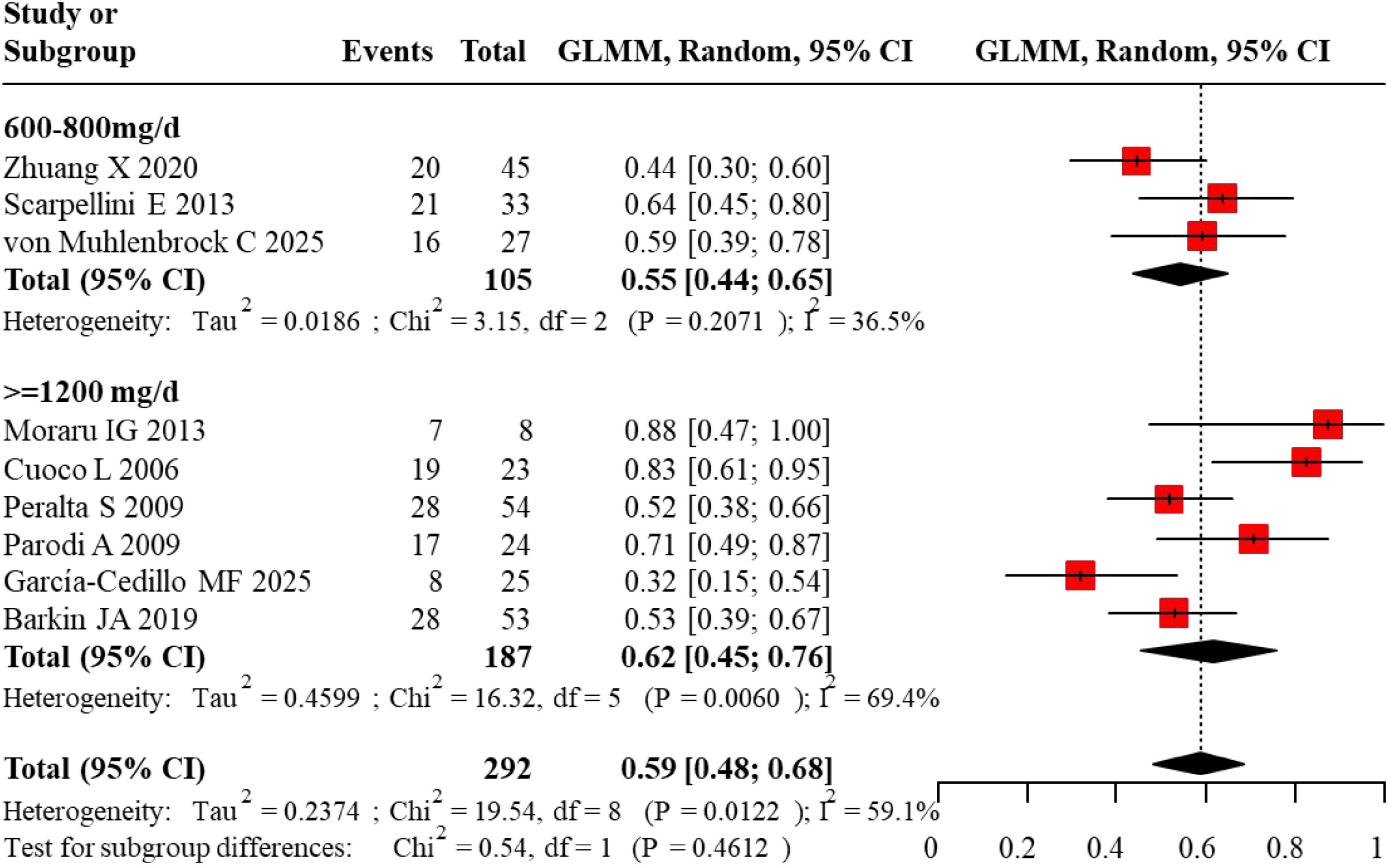
Forest plot for the event rate of SIBO eradication following rifaximin intervention.

The pooled event rate (ER) for SIBO eradication following rifaximin therapy in IBS patients with SIBO was analyzed. Studies were stratified into a lower-dose group (600–800 mg/day) and a medium-to-high-dose group (≥ 1,200 mg/day). In the lower-dose group, individual study ERs ranged from 0.44 to 0.64, with a pooled ER of 0.55 (95% CI: 0.44–0.65). The medium-to-high-dose group showed a wider range of ERs from 0.32 to 0.88, resulting in a pooled ER of 0.62 (95% CI: 0.45–0.76). The overall pooled eradication rate across all doses was 0.59 (95% CI: 0.48–0.68), indicating that approximately 59% of patients achieved SIBO clearance post-treatment in Figure 5.

Additionally, subgroup analysis by treatment duration revealed that the pooled SIBO eradication rate was 64% (95% CI: 0.50–0.76) for the 10-day regimen and 51% (95% CI: 0.43–0.60) for the 14-day regimen. Although the 10-day regimen showed a numerically higher eradication rate, the difference was not statistically significant, and the limited number of studies in the 10-day group (*n* = 2) necessitates caution in interpreting these findings.

**FIGURE 6:**
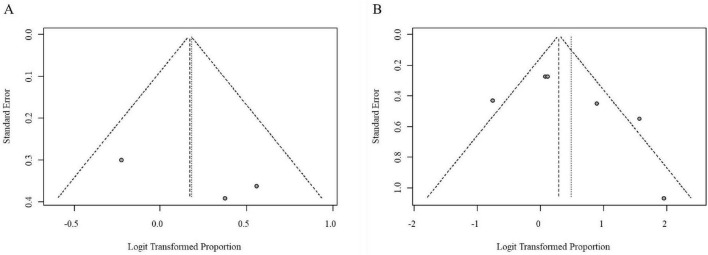
Funnel plots for publication bias assessment in the rifaximin intervention analysis (**A**, Low-dose subgroup; **B**, Medium-to-high-dose subgroup).

Visual inspection of funnel plots for both dose subgroups appeared roughly symmetrical, with data points distributed relatively evenly on either side of the pooled effect estimate, suggesting the absence of substantial publication bias among the rifaximin intervention studies in Figure 6.

**FIGURE 7:**
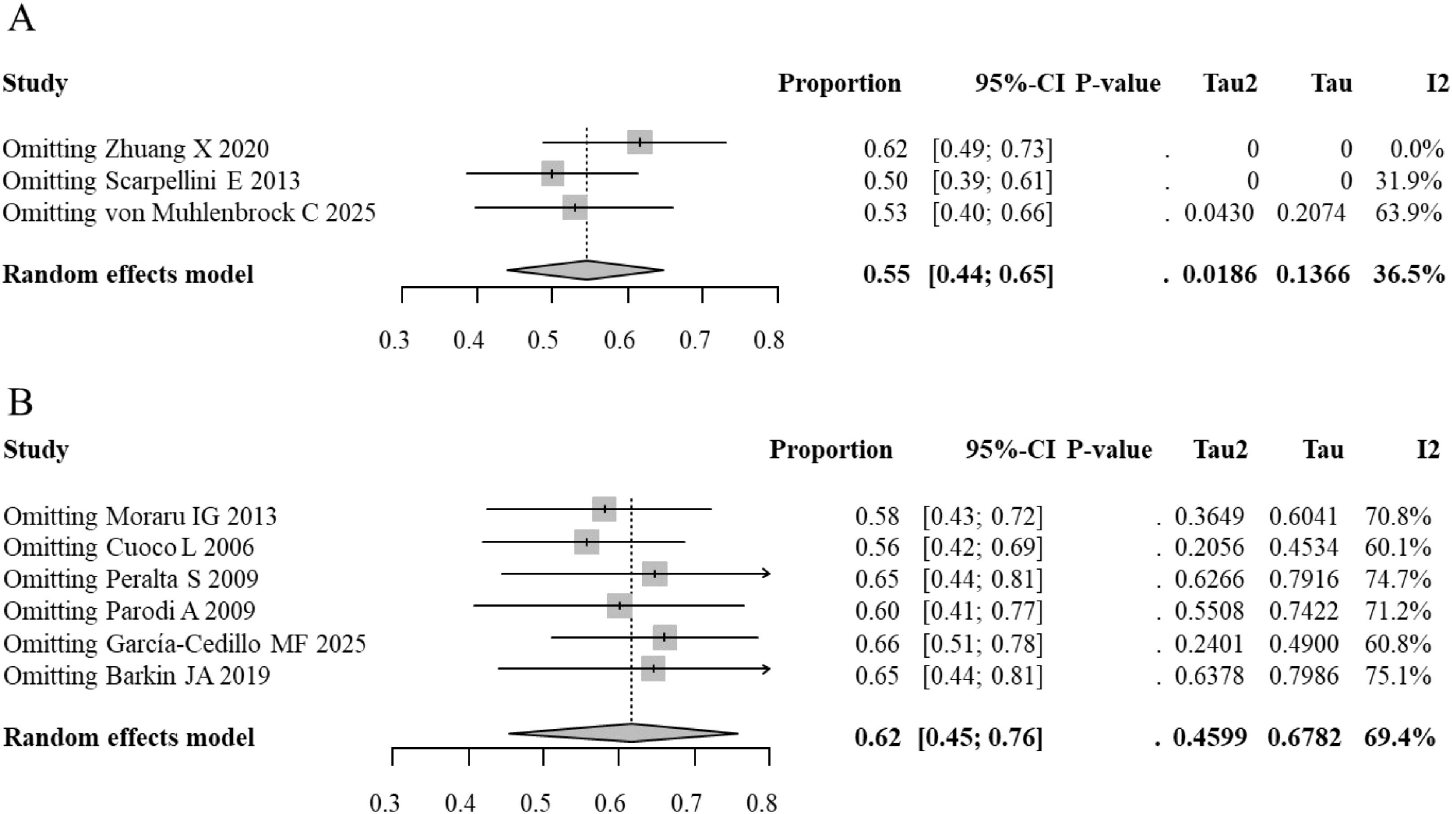
Sensitivity analysis (leave-one-out) forest plots for the event rate of SIBO eradication following rifaximin intervention (**A**, Low-dose subgroup; **B**, Medium-to-high-dose subgroup).

Sensitivity analysis for rifaximin efficacy demonstrated stable results. In the lower-dose group, the pooled ER remained within a narrow range of 0.50–0.62 upon sequential exclusion of studies. Similarly, in the medium-to-high-dose group, the pooled ER varied only slightly from 0.58 to 0.66. The overall significance of the pooled estimate was not altered by the exclusion of any single study, although heterogeneity in the results was found to be influenced by multiple contributing studies in Figure 7.

Regarding microbial gas phenotypes, the included studies did not provide sufficient granular data to perform a formal subgroup analysis comparing eradication rates for hydrogen-predominant SIBO (H*2*-SIBO) versus methane-predominant intestinal methanogen overgrowth (IMO). However, clinical evidence elsewhere suggests that therapeutic responses often diverge by gas type, with methanogens typically exhibiting higher resistance to rifaximin monotherapy compared to hydrogen-producing bacteria. Specifically, recent literature indicates that while rifaximin is highly effective for H*2*-SIBO, methane-positive cases often require dual antibiotic coverage for optimal clearance (e.g., rifaximin combined with neomycin).

Regarding patient-centered outcomes, all nine interventional studies reported improvements in global IBS symptoms, specifically abdominal pain, bloating, and stool consistency. In studies providing categorical data, clinical response rates ranged from 48.1 to 68.0% (Zhuang et al., 2020; Peralta et al., 2009). Notably, the concordance between SIBO eradication and symptom relief was inconsistent across the literature. For example, Scarpellini et al. (2013) found that symptom scores significantly improved only in patients with successful SIBO clearance. In contrast, Zhuang et al. (2020) observed that rifaximin significantly improved symptoms and quality of life (IBS-QOL) regardless of whether SIBO was normalized, suggesting that the clinical benefits of rifaximin may be partially independent of its bactericidal effect on the small bowel (Zhuang et al., 2020).

## 4 Discussion

This systematic review and meta-analysis provides quantitative evidence supporting a significant association between IBS and SIBO and demonstrates the therapeutic utility of rifaximin in this patient subgroup. The findings indicate that IBS patients have a substantially higher risk of SIBO compared to healthy controls (pooled OR = 5.71). Furthermore, treatment with rifaximin resulted in a pooled SIBO eradication rate of 59%, offering high-level evidence to guide clinical management.

The elevated prevalence of SIBO in IBS can be attributed to a complex interplay of pathophysiological mechanisms involving gut motility, microbial ecology, and mucosal integrity. IBS is characterized by dysmotility, which can lead to delayed small intestinal transit, providing an opportunity for bacterial overgrowth. Concurrent alterations in the gut microbiota, including reduced diversity and shifts in predominant phyla, may create a permissive environment for SIBO development (Ding et al., 2017). Additionally, increased intestinal permeability and low-grade mucosal inflammation, commonly observed in IBS, can compromise host defense mechanisms, further facilitating bacterial colonization (Yang et al., 2020). Dysregulation of the gut-brain axis may exacerbate these abnormalities, creating a vicious cycle that sustains both IBS symptoms and SIBO (David et al., 2014). The inclusion of diverse diagnostic criteria (ranging from Rome I to IV and Manning) reflects the evolving landscape of IBS research over the past two decades. Although this contributes to the observed methodological heterogeneity, our sensitivity analysis confirms that the significant association between IBS and SIBO remains robust regardless of the specific diagnostic framework employed (Chu et al., 2016).

Our subgroup analysis based on diagnostic method revealed a slightly higher, more precise association in studies using GBT (pooled OR = 6.09) compared to LBT (pooled OR = 5.14). The GBT subgroup exhibited negligible heterogeneity, suggesting higher diagnostic consistency and specificity for proximal SIBO (Aziz et al., 2017). However, substrate selection represents a fundamental trade-off: GBT’s higher specificity may lead to under-detection (false negatives) of distal SIBO, whereas LBT offers broader sensitivity but is more prone to false positives due to rapid oro-cecal transit (Tang et al., 2024). Rather than suggesting categorical superiority, our findings reinforce the need for context-specific substrate choice: GBT is preferred for confirming proximal SIBO, while LBT may be considered for its distal reach, provided transit time and cut-offs are strictly controlled. The efficacy of rifaximin, a non-absorbable rifamycin derivative, was confirmed in this analysis. Although the difference did not reach statistical significance, a trend toward higher eradication rates was observed with medium-to-high doses (≥ 1,200 mg/day; ER = 0.62) compared to lower doses (ER = 0.55). Regarding treatment duration, our exploratory analysis found no significant difference in efficacy between the 10-day and 14-day regimens, suggesting that current evidence is insufficient to recommend one duration over the other as a superior standard of care. This aligns with prior clinical trials reporting substantial symptom improvement and SIBO clearance with rifaximin. However, a significant limitation of the current literature, including the studies synthesized here, is the lack of routine stratification by gas phenotype (H_2_ vs. CH_4_). Emerging data suggest that the efficacy of rifaximin monotherapy may be significantly lower in patients with methane-predominant IMO compared to those with H*2*-SIBO, as methanogens often necessitate different therapeutic approaches (e.g., combination with neomycin) to achieve comparable eradication rates (Redondo-Cuevas et al., 2024). Its therapeutic advantages are likely multifactorial: Its topical action within the gut lumen reduces pathogenic bacterial loads while minimizing systemic exposure and associated side effects (Deljavan Ghodrati and Comoglu, 2024). Furthermore, beyond its antibacterial effect, rifaximin may modulate the metabolic functions of the gut microbiota and contribute to the restoration of mucosal barrier function (Chedid et al., 2014). Its favorable safety profile and low potential for inducing bacterial resistance make it a suitable agent for managing IBS patients with suspected or confirmed SIBO.

A number of important limitations should be acknowledged when interpreting the findings of this meta-analysis. Firstly, notable clinical and methodological heterogeneity was observed among the included studies, including variations in IBS diagnostic criteria (ranging from Manning and early Rome iterations to Rome IV), different cut-off values for breath tests, and disparate rifaximin dosing regimens and treatment durations. In particular, the limited number of studies specifically comparing 10-day versus 14-day regimens constrained our ability to perform a robust head-to-head evaluation of the optimal treatment length. Although subgroup and sensitivity analyses were performed, these factors may still influence the precision of the pooled estimates. Second, the lack of long-term follow-up data in most included studies precludes conclusions regarding the durability of rifaximin’s effect and SIBO recurrence rates. Third, the inclusion of several single-center studies with relatively small sample sizes raises the possibility of publication bias, as suggested by the asymmetric funnel plot in the LBT subgroup for the association analysis.

## 5 Conclusion

This meta-analysis strengthens the evidence for a significant link between IBS and SIBO and substantiates the role of rifaximin as an effective treatment. Our analysis highlights that diagnostic substrate selection is a trade-off between the high specificity of GBT and the broad sensitivity of LBT, suggesting that clinical protocols should anchor substrate choice to the specific diagnostic context. Despite existing limitations related to study heterogeneity and diagnostic variability, the findings provide a robust evidence base to inform clinical practice. Future research should prioritize large-scale, well-designed trials employing standardized diagnostic protocols to further refine patient selection (including gas phenotype stratification), optimize treatment regimens tailored to specific microbial signatures (e.g., H*2*-SIBO vs. IMO), and evaluate the long-term outcomes, and evaluate the long-term outcomes of targeting SIBO in the management of IBS.

## Data Availability

The original contributions presented in the study are included in the article/Supplementary material, further inquiries can be directed to the corresponding author.
